# Deciphering Human Heat Shock Transcription Factor 1 Regulation via Post-Translational Modification in Yeast

**DOI:** 10.1371/journal.pone.0015976

**Published:** 2011-01-06

**Authors:** Liliana Batista-Nascimento, Daniel W. Neef, Phillip C. C. Liu, Claudina Rodrigues-Pousada, Dennis J. Thiele

**Affiliations:** 1 Genomics and Stress Laboratory, Instituto de Tecnologia Química e Biológica, Oeiras, Portugal; 2 Department of Pharmacology and Cancer Biology, Duke University School of Medicine, Durham, North Carolina, United States of America; 3 Applied Technology Group, Incyte Corporation, Wilmington, Delaware, United States of America; University of Kent, United Kingdom

## Abstract

Heat shock transcription factor 1 (HSF1) plays an important role in the cellular response to proteotoxic stresses. Under normal growth conditions HSF1 is repressed as an inactive monomer in part through post-translation modifications that include protein acetylation, sumoylation and phosphorylation. Upon exposure to stress HSF1 homotrimerizes, accumulates in nucleus, binds DNA, becomes hyper-phosphorylated and activates the expression of stress response genes. While HSF1 and the mechanisms that regulate its activity have been studied for over two decades, our understanding of HSF1 regulation remains incomplete. As previous studies have shown that HSF1 and the heat shock response promoter element (HSE) are generally structurally conserved from yeast to metazoans, we have made use of the genetically tractable budding yeast as a facile assay system to further understand the mechanisms that regulate human HSF1 through phosphorylation of serine 303. We show that when human HSF1 is expressed in yeast its phosphorylation at S303 is promoted by the MAP-kinase Slt2 independent of a priming event at S307 previously believed to be a prerequisite. Furthermore, we show that phosphorylation at S303 in yeast and mammalian cells occurs independent of GSK3, the kinase primarily thought to be responsible for S303 phosphorylation. Lastly, while previous studies have suggested that S303 phosphorylation represses HSF1-dependent transactivation, we now show that S303 phosphorylation also represses HSF1 multimerization in both yeast and mammalian cells. Taken together, these studies suggest that yeast cells will be a powerful experimental tool for deciphering aspects of human HSF1 regulation by post-translational modifications.

## Introduction

All organisms are exposed to proteotoxic stresses that result in the accumulation of misfolded proteins. In response to these stresses cells have evolved adaptive responses to protect and stabilize cellular proteins until more favorable conditions for cell proliferation are encountered [1]. The heat shock transcription factor, HSF, is a homotrimeric transcription factor that activates gene expression in response to a variety of stresses including heat and oxidative stress, as well as inflammation and infection [2]. Recent evidence has shown that the *S. cerevisiae* HSF directly activates the expression of genes whose protein products are involved in protein folding and degradation, ion transport, signal transduction, energy generation, carbohydrate metabolism, vesicular transport, cytoskeleton formation and other cellular functions [3].

While mammalian cells express four distinct HSF proteins encoded by separate genes, HSF1 is the primary factor responsible for stress responsive gene transcription [2]. In the absence of stress, mammalian HSF1 is repressed through mechanisms that are not well understood. HSF1 is thought be maintained in an inactive monomeric state through intramolecular interactions between a hydrophobic coiled-coil domain in the carboxyl-terminus of the protein and three amino-terminal coiled-coils required for homotrimerization and transcriptional activation [4], [5], [6]. HSF1 is also thought to be bound and repressed by the protein chaperones Hsp90 and Hsp70, though it is not clear how these chaperones repress HSF1 activity [7], [8], [9], [10]. Studies suggest that during the initial phase of the stress response, the inactive HSF1 monomer dissociates from Hsp90, homotrimerizes, is transported to the nucleus and binds to heat shock elements (HSE) found in the promoters of HSF target genes [10], [11]. The DNA-bound homotrimer, remains relatively transcriptionally inert [12], potentially due to the continued interaction with Hsp70 and the HSF1-transactivation domain [9]. Stress-dependent hyperphosphorylation of HSF1 by potentially multiple protein kinases has been proposed to, in part, promote HSF1 dependent transactivation [13], [14], [15].

The activity of HSF1 is also thought to be negatively regulated through a number of post-translational modifications including phosphorylation, sumoylation and acetylation [16], [17], [18], [19]. Mass spectrometry analyses have shown HSF1 to be phosphorylated on at least 12 serine residues [13] and phosphorylation of S121, S303, S307 and S363 have been correlated with a repression in HSF1 activity [18], [20], [21]. The most comprehensively studied of these phosphorylation events are the phosphorylation of S303 and S307. However, much of what is known about S303 and S307 phosphorylation stems from *in vitro* phosphorylation experiments and *in vivo* studies using either lexA or Gal4-HSF1 fusion proteins lacking the native HSF1 DNA binding domain. As such, many of the earlier studies exploring S303 and S307-dependet regulation of HSF1 activity have resulted in conflicting results. For example, previous phosphorylation experiments suggested that S307 was phosphorylated by ERK which, in turn, acted as an essential priming step for GSK3-dependent phosphorylation of S303 [22]. However, subsequent *in vitro* studies suggested that S303 could also be phosphorylated by a variety of mitogen activated protein kinases (MAPK) including the stress responsive MAPK p38 [17], [18]. In addition, subsequent *in vivo* data suggested S303 phosphorylation could occur independently of S307 phosphorylation [16].

While the specific mechanism by which S303 and S307 phosphorylation repress HSF1 activity remains unclear, evidence has suggested that S303 and S307 phosphorylation represses the transactivation potential of HSF1 [18], [22], [23]. S303 and S307 are constitutively phosphorylated in the absence of stress and S303 phosphorylation levels increase after exposure to stress, suggesting that this phosphorylation event might also contribute to HSF1 inactivation during the recovery phase [16], [17]. Interestingly, phosphorylation of S303, but not S307, promotes sumoylation of K298 [16] which, like S303 phosphorylation, also increases in response to stress exposure and represses HSF1-dependent transactivation [24]. However, it remains unclear if the repressive effects of S303 phosphorylation on HSF1 activity are exclusively mediated through K298 sumoylation or occur through additional mechanisms.

While HSF1 and the cognate HSEs are quite well conserved from yeast to humans, our previous results demonstrated that human HSF1 expressed in *S. cerevisiae* is unable to complement for the loss of the essential yeast HSF protein [25]. Further analysis showed that human HSF1 expressed in yeast was unable to form a homotrimer and consequently unable to activate HSE-dependent gene expression to support cell viability. Human HSF1 homotrimerized, became active and complemented for the loss of yeast HSF when three derepressing mutations, collectively known as LZ4m, were introduced into the repressive carboxyl-terminal coiled-coil domain [6], [25]. Further studies in yeast identified an amino-terminal linker-domain as well as a loop in the DNA binding domain as repressive elements that contributed to HSF1 repression in both yeast and mammalian cells [26], [27]. We have also used to the yeast assay system to screen for and indentify novel pharmacological activators of human HSF1 [28]. Together, these results suggest that human HSF1 expressed in yeast is maintained in a constitutively repressed state through mechanisms similar to those of mammalian cells and that the yeast system can serve as a simplified assay system to decipher the complex mechanisms regulating human HSF1 activity.

Here we report the use of the yeast assay system to further understand the mechanisms that regulate human HSF1 through phosphorylation of serine 303. Our results suggest that S303 phosphorylation blocks human HSF1 homotrimerization thereby preventing human HSF1 activation and complementation of the loss of yeast HSF. Furthermore, we demonstrate that S303 phosphorylation also blocks HSF1 homotrimerization in mammalian cells. We show that phosphorylation of HSF1 S303 in yeast occurs via the action of the MAPK Slt2 and not via the action of GSK3 and we extend these findings to show that S303 phosphorylation also occurs independent of GSK3 in mammalian cells.

## Results

### Phosphorylation of S303 contributes to repression of human HSF1 in yeast

**Figure 1:**
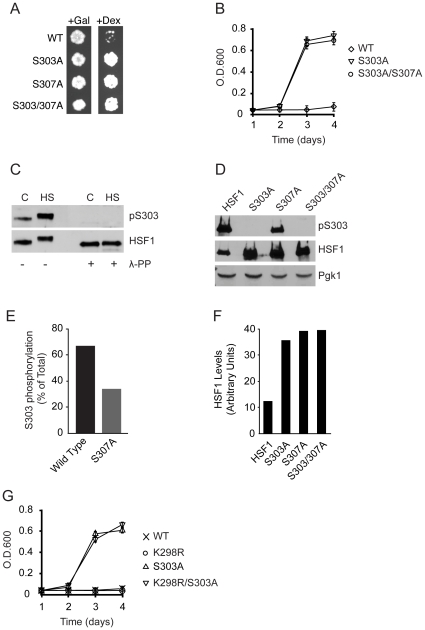
S303 phosphorylation represses HSF1 activity in yeast. (**A**) PS145 yeast strains expressing wild-type HSF1 (WT) or the S303A, S307A or S303/307A mutants were plated on either galactose or dextrose supplemented medium. (**B**) PS145 expressing either wild-type HSF1 or the S303A or S303/307A mutants were grown in dextrose containing medium for 4 d. Growth was monitored by measuring O.D._600_. (**C**) HeLa cells were grown at 37°C (C) or heat shocked for 2 h at 42°C (HS). Total protein extracts were treated with lambda protein phosphatase and analyzed for phospho-S303 (pS303) and total HSF1 levels by immunoblotting. (**D**) PS145 was transformed with a plasmid expressing wild-type HSF1 (WT) or mutant alleles of HSF1 and grown on galactose containing medium. Total protein extracts were analyzed for pS303, HSF1 and Pgk1 by immunoblotting. (**E**) Levels of HSF1 phosphorylated at S303 were quantified and are shown as a percent of total HSF1, from panel D. (**F**) Protein levels of HSF1 were normalized to Pgk1, from panel D. (**G**) PS145 expressing either wild-type HSF1 or mutant HSF1 alleles were assayed for HSF1-dependent growth as in **B**.

When human HSF1 is expressed in yeast it is unable to homotrimerize, promote gene expression and complement for the loss of the essential yeast HSF protein [25]. Because our previous work suggested that when HSF1 is expressed in yeast it exists in a constitutively repressed monomeric state, we sought to use the yeast assay system to better understand the complex mechanisms regulating HSF1 activity in mammalian cells. An important component of HSF1 repression occurs through the phosphorylation of serine 303 and serine 307 [17], [18]. Because S303 and S307 are constitutively phosphorylated in mammalian cells and alanine substitution of S303 or S307 promotes constitutive activation of HSF1 in mammalian cells in reporter assays [17], [18] we tested whether S303 and/or S307 contribute to HSF1 repression in yeast. Wild-type HSF1 or the individual S303A, S307A or S303/307A double mutants were expressed in yeast strain PS145 which lacks a chromosomal copy of the essential yeast HSF gene and constitutively expresses yeast HSF episomally from a galactose inducible and dextrose repressible promoter [29]. When PS145 is grown in the presence of dextrose as the sole carbon source, yeast HSF expression is extinguished and growth becomes solely dependent on HSF1 which is episomally expressed [28]. While wild-type human HSF1 was unable to complement for the loss of yeast HSF, expression of the S303A, S307A or S303/307A HSF1 mutants allowed for human HSF1-dependent yeast growth (Figure 1A, B). Interestingly, the S303/307A double HSF1 mutant did not display enhanced activity over the S303A mutant (Figure 1B) suggesting that phosphorylation of both S303 and S307 modulate HSF1 repression through similar mechanisms.

To ascertain whether HSF1 is being phosphorylated in yeast, we employed a commercially available antibody specific for phospho-S303 (pS303). Because this antibody has not previously been characterized in the literature, we tested its specificity in human HeLa cells where S303 is known to be constitutively phosphorylated [17]. As shown in Figure 1C, using this antibody we detected that endogenous HSF1 was constitutively phosphorylated in HeLa cells in the absence of stress. We also observed an increase in S303 phosphorylation in response to a heat shock, which correlated with previous reports [16]. Importantly, the pS303-specific antibody did not detect HSF1 when HeLa extracts were treated with lambda protein phosphatase prior to immunoblot analysis (Figure 1C), nor does it detect HSF1 when S303 is mutated to alanine (Figure 1D). Together, these data suggest that the antibody is specific for HSF1 that is phosphorylated on S303. The detection of HSF1 using a polyclonal anti-HSF1 antibody demonstrates that there are no significant differences in the steady state levels of HSF1 either treated or untreated with lambda phosphatase (Figure 1C).

Consistent with a contribution to HSF1 repression (Figure 1A, B) S303 is robustly phosphorylated when HSF1 is expressed in yeast (Figure 1D, E). Interestingly, phosphorylation of S303 was also observed when the S307A mutant was expressed in yeast though it was reduced by approximately 50% when compared to wild-type HSF1 (Figure 1D, E). While this observation supports a previous report indicating that S303 phosphorylation could occur independently of S307 phosphorylation in mammalian cells [16], these data also suggests that under certain circumstances S303 phosphorylation may be enhanced by S307 phosphorylation. In addition, although a correlation between S303 and S307 phosphorylation and HSF1 protein stability has not been previously reported, we repeatedly observed two to three-fold higher steady state levels of HSF1 when the S303A, S307A and S303/307A mutants were expressed in yeast (Figure 1D, F). While an antibody specific for phospho-S307 is commercially available, we have been unable to detect S307 phosphorylation of human HSF1. As such we focused our investigation on S303-phosphorylation dependent repression of human HSF1.

Human HSF1 S303 phosphorylation is known to promote sumoylation of lysine 298, which also contributes to the repression of HSF1 activity [16]. Therefore, to further investigate the idea that human HSF1 is being actively repressed in yeast, we explored the possibility that K298, like S303, contributes to HSF1 repression in yeast. However, unlike the S303A HSF1 mutant, the K298R mutant did not promote HSF1-dependent growth (Figure 1E), suggesting that at least in yeast, K298 does not significantly contribute to HSF1 repression. We also did not observe a reduction in human HSF1-dependent yeast growth for the S303A/K298R double mutant, indicating that K298 is also not required for HSF1 activity in yeast (Figure 1E).

### S303 represses trimer formation of HSF1 in yeast and mammalian cells

**Figure 2:**
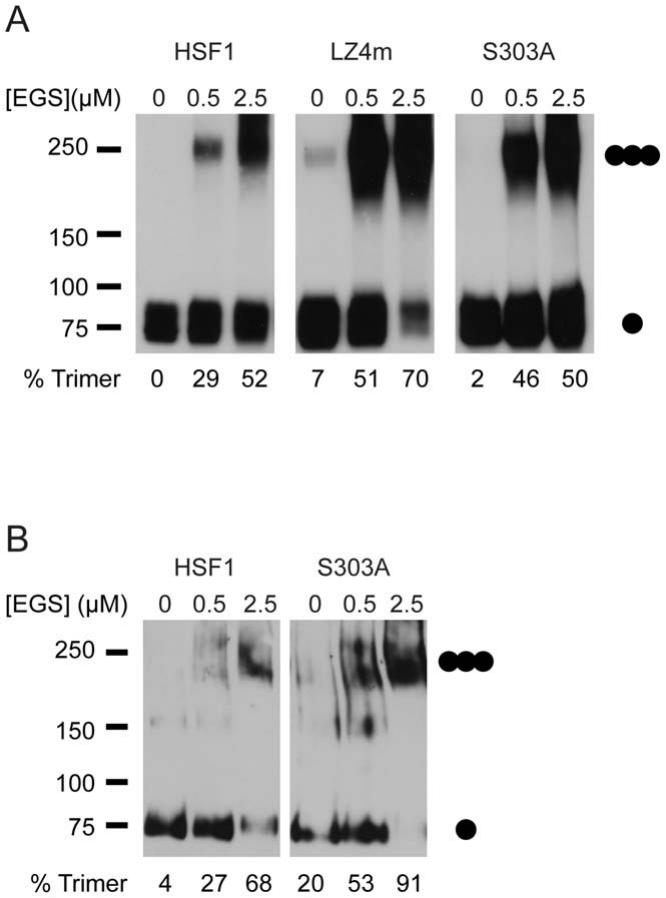
S303 represses trimer formation of HSF1 in yeast and mammalian cells. (**A**) PS145 was transformed with wild-type HSF1, the LZ4m mutant or the S303A mutant and grown on galactose containing medium. Total protein extracts were evaluated for HSF1 multimerization by EGS crosslinking, SDS-PAGE, and immunoblotting using an HSF1 specific antibody. The positions of molecular weight markers are indicated on the left, and circles indicating the expected migration of HSF1 monomers and trimers are on the right. Levels of HSF1 trimer as percent of total HSF1 are shown below. (**B**) hsf1^−/−^ MEFs were transfected with a plasmid expressing wild-type HSF1 or the S303A mutant and analyzed for HSF1 multimerization by EGS cross-linking as in A.

Previous reports have suggested that phosphorylation of S303 represses the ability of HSF1 to transactivate gene expression [18], [22], [23]. Here we show that the HSF1 S303A mutant functionally complements for the lost of yeast HSF (Figure 1A, B). Based on our previous work this indicates that S303 phosphorylation might also regulate the ability of human HSF1 to homotrimerize [25]. To test this hypothesis we carried out EGS cross-linking experiments in conjunction with immunoblot analysis to ascertain if S303 phosphorylation regulates the homotrimerization of human HSF1 in yeast. When the S303A HSF1 mutant was expressed in yeast we detected approximately 2-fold higher levels of trimerized HSF1 at the intermediate EGS concentration than when wild-type HSF1 was expressed in yeast (Figure 2A). However, trimerization of the S303A HSF1 mutant was lower than trimerization of the LZ4 HSF1 mutant, previously demonstrated to be constitutively trimerized in yeast and mammalian cells and able to complement for the loss of yeast HSF [6], [25]. We observed similar results for the S307A and S303/307A HSF1 mutants (data not shown) further supporting the notion that S303 and S307 phosphorylation repress HSF1 activity through similar mechanisms. We next evaluated whether HSF1 S303 phosphorylation could also function to repress homotrimer formation in mammalian cells. To test this hypothesis we expressed wild-type HSF1 or the S303A, S307A or S303/307A mutants in hsf1^−/−^ mouse embryonic fibroblasts (MEF) [30] and assayed for HSF1 trimerization in the absence of thermal stress by EGS crosslinking and immunoblotting. While wild type HSF1 could be detected as a multimer in these extracts, we observed approximately 2-fold higher levels of the HSF1 trimer for the HSF1 S303A mutant (Figure 2B) as well as the S307A and S303/307A mutants (data not shown).

### S303 phosphorylation and coiled-coil interactions synergize in HSF1 repression

**Figure 3:**
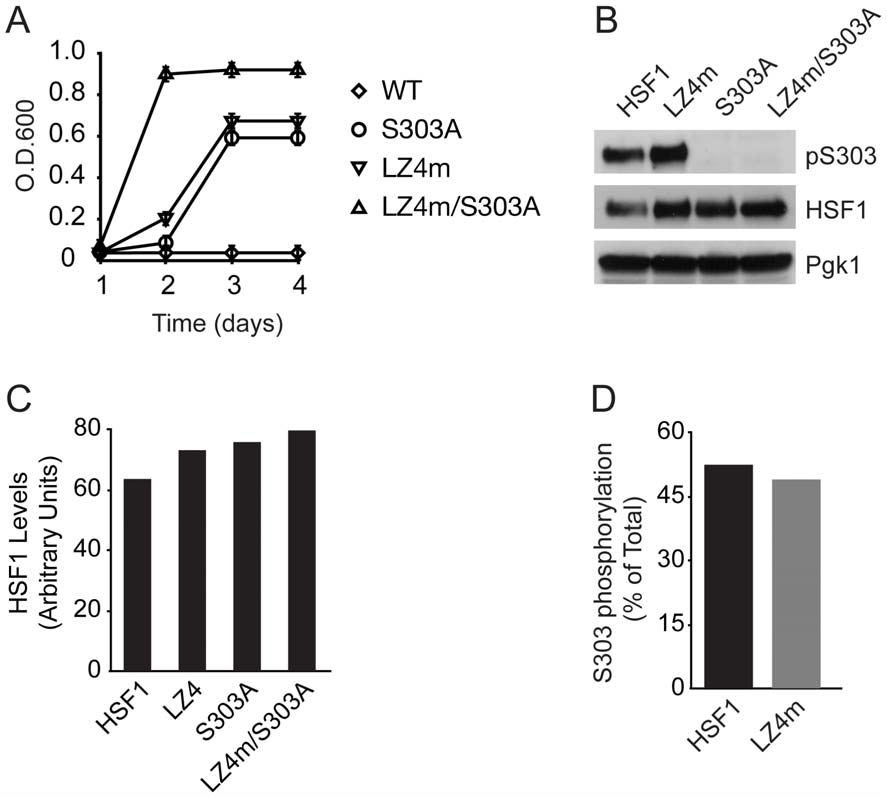
Phosphorylation of S303 and coiled-coil domains synergize in the repression of HSF1 in yeast. (**A**) PS145 expressing either wild-type HSF1 or mutant alleles of HSF1 were grown in dextrose supplemented medium for 4 d. Growth was monitored by measuring O.D._600_. (**B**) PS145 was transformed with a plasmid expressing wild-type HSF1 (WT) or mutant alleles of HSF1 and grown on galactose containing medium. Total protein extracts were analyzed for pS303, total HSF1 and Pgk1 by immunoblotting. (**C**) Protein levels of HSF1 were normalized to Pgk1, from panel B. (**D**) Levels of HSF1 phosphorylated at S303 were quantified and are shown as a percent of total HSF1, from panel B.

In addition to post-translational modifications, HSF1 activity is also thought to be repressed through intramolecular interactions between carboxyl- and amino-terminal coiled-coil domains and mutations in these domains render HSF1 constitutively trimerized, nuclear localized and bound to DNA in mammalian cells [6]. Because our results suggest that S303 phosphorylation might also regulate homotrimer formation, we tested the combined affects of both the S303A as well as the LZ4m mutations on human HSF1 activity in yeast. A human HSF1 mutant containing both the S303A and LZ4m mutations was created and its ability to promote human HSF1-dependent yeast growth was compared to the individual HSF1 mutants as well as wild-type HSF1 in quantitative cell growth assays. The individual S303A and LZ4m HSF1 mutants promoted human HSF1-dependent yeast growth to a similar extent, though neither the LZ4m nor the S303 mutant were fully derepressed, as the S303A/LZ4m double mutant displayed enhanced human HSF1-dependent yeast growth (Figure 3A). While we currently do not know if the S303A/LZ4m double HSF1 mutant has an increased propencity to trimerize, previous studies have shown that the LZ4m mutant, when expressed in yeast is not maximally trimerized and trimerization can be further enhanced via the addition of pharmacological HSF1 activators [28]. While we observed higher steady state protein levels for both the S303A and LZ4m mutants in comparison to wild-type HSF1 when expressed in yeast, no further increases in protein levels were observed for the double mutant (Figure 3B, C). These results suggest that while both HSF1 S303 phosphorylation and coiled-coil interactions regulate human HSF1 multimerization in yeast, they do so via distinct mechanisms. We also did not observe changes in HSF1 S303 phosphorylation when the LZ4m mutant was expressed in yeast, consistent with the notion that HSF1 trimerization does not affect HSF1 S303 phosphorylation.

### Gsk3 regulates human HSF1 activity in yeast independent of S303 phosphorylation

**Figure 4:**
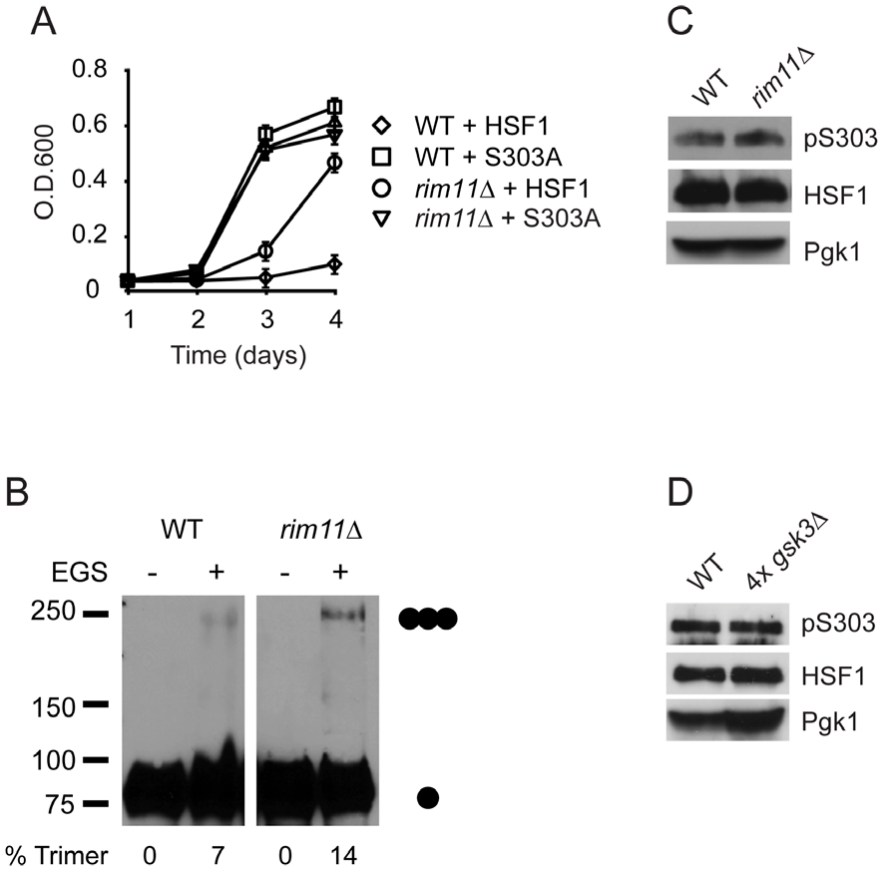
GSK3 represses HSF1 activity in yeast independent of S303. (**A**) PS145 (WT) expressing wild-type HSF1 or the S303A HSF1 mutant and LNY1 (*rim11*Δ) expressing wild-type HSF1 were grown in dextrose supplemented medium for 4 d. Growth was monitored by measuring O.D._600_. (**B**) PS145 (WT) and LNY1 (*rim11*Δ) expressing wild-type HSF1 were grown on galactose containing medium and were evaluated for HSF1 multimerization by EGS crosslinking, SDS-PAGE, and immunoblotting using an HSF1 specific antibody. The positions of molecular weight markers are indicated on the left, and circles indicating the expected migration of HSF1 monomers and trimers are on the right. Levels of HSF1 trimer as percent of total HSF1 are shown below. (**C**) PS145 (WT) and LNY1 (*rim11*Δ) were transformed with a plasmid expressing wild-type HSF1 and were grown on galactose containing medium. Total protein extracts were analyzed for pS303, total HSF1 and Pgk1 by immunoblotting. (**D**) YPH499 (WT) and LNY3 (4x*gsk3*Δ) were transformed with a plasmid expressing wild-type HSF1 and were grown in dextrose containing medium. Total protein extracts were analyzed for pS303, total HSF1 and Pgk1 by immunoblotting.

Previous reports using *in vitro* phosphorylation experiments have suggested that HSF1 is phosphorylated at S303 by glycogen synthase kinase 3 (GSK3) [20], [22], [31]. However, it remains unclear if GSK3 phosphorylates and represses HSF1 via S303 phosphorylation *in vivo*. To test if GSK3 contributes to HSF1 repression, we assayed human HSF1-dependent yeast growth in a strain also lacking the yeast GSK3 homolog Rim11. Supporting the notion that yeast GSK3 can repress human HSF1 activity in yeast we observed human HSF1-dependent yeast growth as well as HSF1 multimerization in the *rim11*Δ strain (Figure 4A, B). However, HSF1-dependent yeast growth in the *rim11*Δ strain was less robust than growth of a wild-type strain expressing the S303A HSF1 mutant (Figure 4A). Furthermore, when we expressed the S303A HSF1 mutant in the *rim11*Δ strain we observed HSF1-dependent growth at a rate similar to the growth of the S303A mutant in wild-type cells. This suggested the possibility that HSF1 might not be fully derepressed in the *rim11*Δ strain. Consistent with this idea, we did not detect a reduction in S303 phosphorylation in the *rim11*Δ strain (Figure 4C). *S. cerevisiae* encodes four separate yet partially functionally redundant GSK3 homologues [32], suggesting the possibility that S303 remains phosphorylated in the *rim11*Δ strain due to phosphorylation through other GSK3 proteins. To test this hypothesis we assayed the phosphorylation state of HSF1 at S303 in a yeast strain lacking all four isoforms of yeast GSK3. As shown in Figure 4D, no reduction in S303 phosphorylation was observed in the 4x*gsk3*Δ strain suggesting that while yeast GSK3 does contribute to HSF1 repression, it does so independently of S303 phosphorylation.

**Figure 5:**
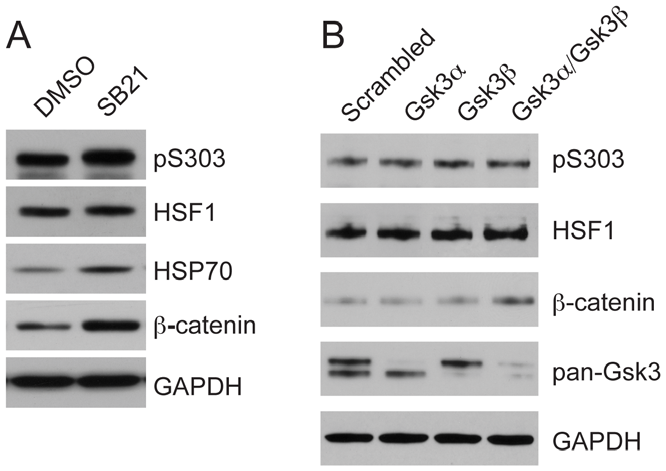
GSK3 represses HSF1 activity in HeLa cells independent of S303 phosphorylation. (**A**) HeLa cells were treated with DMSO solvent or the GSK3 inhibitor SB-216763 (25 µM) for 15 h. Total protein was analyzed for pS303, HSF1, and β-catenin by immunoblotting. GAPDH serves as a loading control. (**B**) HeLa cells were treated with siRNA specific for GSK3α and GSK3β either individually or together or a scrambled siRNA for 72 h. Total protein was analyzed for pS303, total HSF1, β-catenin, GSK3α/β and GAPDH by immunoblotting.

Results shown here for the S303A HSF1 mutant and previously published for the LZ4m HSF1 mutant suggest that mechanisms that regulate HSF1 in mammalian cells are at least partially conserved with regulation of human HSF1 expressed in yeast cells. Therefore, we carried out experiments to ascertain if GSK3 might also repress HSF1 independently of S303 phosphorylation in mammalian cells. To explore this possibility HeLa cells were treated with the GSK3 inhibitor SB-216763 [33] and assayed for HSF1 S303 phosphorylation as ascertained by immunoblotting with the anti-pS303 antibody. While SB-216763 treatment strongly inhibited GSK3 activity as shown by increased β-catenin levels [34], no reduction in S303 phosphorylation was observed (Figure 5A). However, similar to the results obtained from our yeast experiments, SB-216763 did promote activation of HSF1 under normal growth conditions, as determined by immunoblot analysis of Hsp70 expression (Figure 5A). This result is consistent with a previous report showing increased Hsp70 expression in response to lithium treatment, which also inhibits GSK3 function [35]. siRNA mediated knock-down of the two GSK3 isoforms in mammals, GSK3α and GSK3β, either singly or in combination, further confirmed that, while β-catenin expression was elevated, HSF1 S303 was not appreciably phosphorylated by GSK3 in unstressed mammalian cells (Figure 5B). Together, data from experiments in both yeast and mammalian cells support a model in which GSK3 inhibits HSF1 activity through a mechanism that is independent of S303 phosphorylation.

### Slt2 represses human HSF1 activity via S303 phosphorylation in yeast

**Figure 6:**
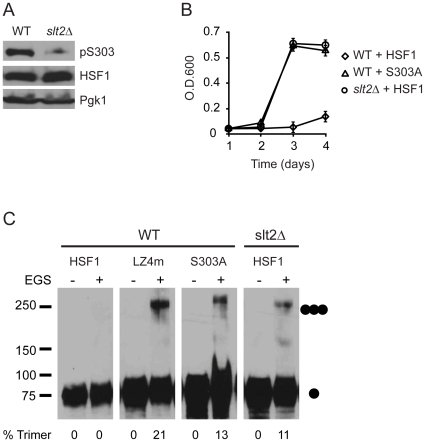
S303 phosphorylation of HSF1 in yeast is modulated by Slt2. (**A**) PS145 and LNY2 (*slt2*Δ) were transformed with a plasmid expressing wild-type HSF1 and were grown on galactose containing medium. Total protein extracts were analyzed for pS303, total HSF1 and Pgk1 by immunoblotting. (**B**) PS145 (WT) expressing wild-type HSF1 or the S303A mutant or LNY2 (*slt2*Δ) expressing wild-type HSF1 were grown in dextrose supplemented medium for 4 d. Growth was monitored by measuring O.D._600_. (**C**) PS145 (WT) expressing wild-type HSF1, the LZ4m mutant or the S303A mutant and LNY2 (*slt2*Δ) expressing HSF1 were evaluated for HSF1 multimerization by EGS cross-linking, SDS-PAGE, and immunoblotting. The positions of molecular weight markers are indicated on the left and circles indicating the expected migration of HSF1 monomers and trimers are on the right. Levels of HSF1 trimer as percent of total HSF1 are shown below.

To begin to identify which protein kinase(s) in yeast phosphorylate human HSF1 at S303 to promote HSF1 repression, we assayed S303 phosphorylation in several previously generated protein kinase deletion strains obtained from the yeast gene deletion collection [36]. One strain in which we detected severely reduced levels of human HSF1 S303 phosphorylation was a strain deleted for the *SLT2* gene, encoding a stress-responsive MAPK [37], [38], consistent with S303 lying within a consensus site for MAPK-dependent phosphorylation (Figure 6A) [39]. This suggests that Slt2 either directly or indirectly promotes the phosphorylation of human HSF1 expressed in yeast. This hypothesis was further supported by the observation that an *slt2*Δ strain allowed wild type human HSF1-dependent yeast growth at a rate similar to the HSF1 S303A mutant, while no growth was observed in the *SLT2* wild-type strain (Figure 6B). Homotrimerization of wild-type human HSF1 was observed in the *slt2*Δ strain at levels similar to the S303A and LZ4m HSF1 mutants, further supporting the notion that the Slt2 MAPK represses human HSF1 multimerization in yeast (Figure 6C). In mammalian cells the most closely related homolog of Slt2 is the MAPK ERK5 [40]. However, using siRNA-mediated knock-down of ERK5 we were unable detect an effect of ERK5 on HSF1 S303 phosphorylation in mammalian cells (data not shown). This may suggest that in mammalian cells S303 can be phosphorylated by multiple MAPKs. This hypothesis is supported by previous data showing that ERK1/2 as well as the stress-responsive MAPK p38 could phosphorylate HSF1 at S303 *in vitro* [18]. In addition, our data showing reduced, but not eliminated phosphorylation of S303 in the *slt2*Δ strain (Figure 6A) also support a model where S303 may be phosphorylated by multiple MAPKs.

### Expression of S303A and S307A mutants in hsf1^−/−^ cells results in constitutive activation of Hsp70 expression

**Figure 7:**
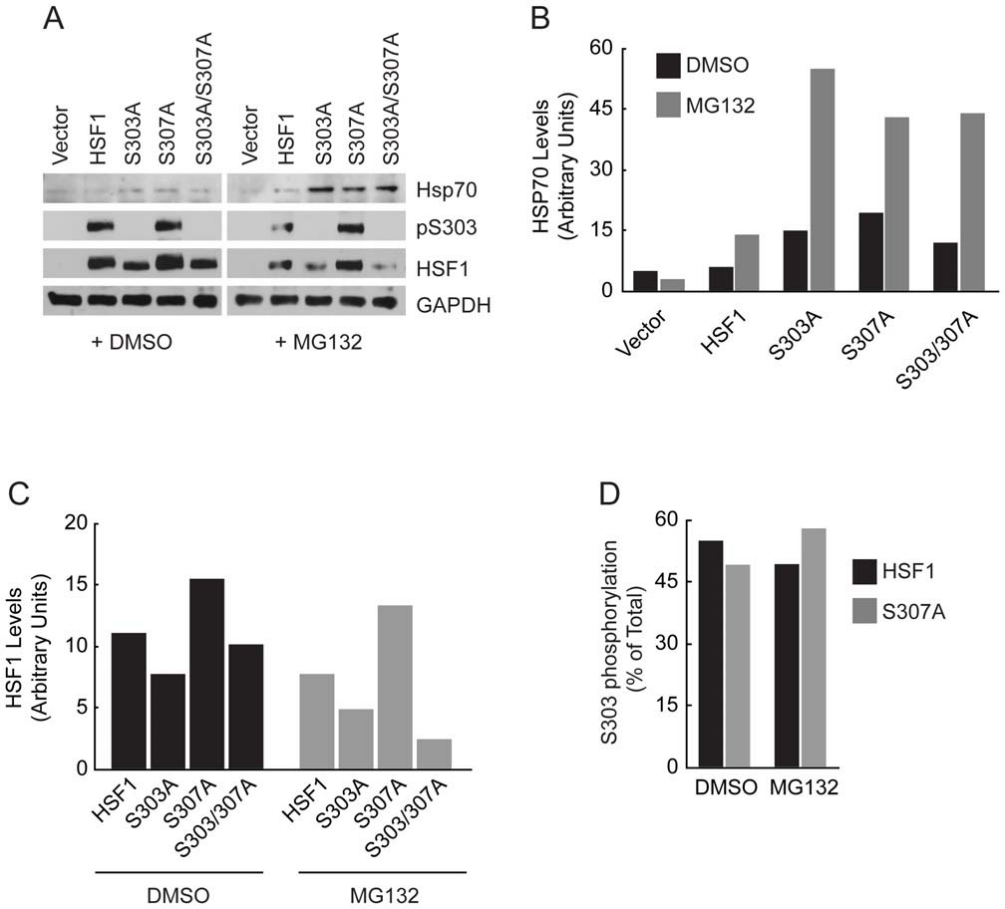
S303 and S307 repress HSF1 activity in hsf1^−/−^ MEFs. (**A**) hsf1^−/−^ MEFs were transfected with an empty vector or plasmids expressing wild-type HSF1 or the S303A, S307A or the S303/307A mutants. The transfected cells were treated with DMSO solvent or MG132 (10 µM) for 5 h. Total protein extracts were analyzed for Hsp70, pS303 and HSF1 by immunoblotting. GAPDH serves as a loading control. (**B**) Protein levels of Hsp70 were normalized to GAPDH, from panel A. (**C**) Protein levels of HSF1 were normalized to GAPDH, from panel A. (**D**) Levels of HSF1 phosphorylated at S303 were quantified and are shown as a percent of total HSF1, from panel A.

Previous studies have assayed the function of S303 and S307 phosphorylation in HSF1 regulation via *in vitro* phosphorylation experiments [22], *in vivo* using lexA/Gal4-HSF1 fusion proteins lacking the native HSF1 DNA binding domain [17], [18] or via overexpression of a S303A HSF1 mutants in mammalian cells expressing endogenous wild-type HSF1 [16]. We tested the consequences of loss of S303 and S307 phosphorylation on HSF1 activity in the context of the entire protein using hsf1^−/−^ MEFs which lack endogenous HSF1. When we expressed S303A, S307A or S303/307A HSF1 mutants in hsf1^−/−^ MEFs we observed a modest elevation of Hsp70 expression under normal growth conditions (Figure 7A, B) consistent with the hypothesis that S303 phosphorylation modulates both homotrimerization as well as transactivation by HSF1. However, HSF1 was not fully activated through the S303A and S307A mutations, as expression of Hsp70 was further enhanced when the transfected cells were exposed to low levels of the proteasome inhibitor MG132 (Figure 7A, B). This is consistent with our data generated in yeast demonstrating that while the S303A mutation did activate human HSF1-dependent yeast growth, this was further enhanced when the S303A HSF1 mutant was combined with the LZ4m mutation (Figure 3A). Interestingly, in hsf1^−/−^ cells we observed a faster electrophoretic mobility on SDS-PAGE gels for the HSF1 S303A and S303/307A mutant proteins that was not observed for wild-type HSF1 or the S307A mutant (Figure 7A), nor did we observe this change in mobility in the yeast system (Figure 1D). While the nature of this electrophoretic mobility shift is unknown, the HSF1 S303A and S303/S307A mutant alleles also exhibited lower steady state levels when exposed to MG132, suggesting that these proteins, despite having increased activity, might be less stable (Figure 7A, C). Because S303 phosphorylation has been proposed to promote HSF1 sumoylation in mammalian cells [16] it is possible that lack of sumoylation results in the altered electrophoretic mobility. Despite the fact that equal amounts of plasmid DNA were transfected for each mutant, we observed elevated steady state protein levels for the HSF1 S307A mutant (Figure 7A, C). While we have not definitively demonstrated that the S307A mutant protein has increased stability in comparison to wild-type HSF1, this finding correlates with the increased protein levels we observed for the HSF1 mutants expressed in yeast (Figure 1D, F) and will require further investigation. Interestingly, when we expressed the HSF1 S307A mutant in hsf1^−/−^ cells we did not observe a reduction in S303 phosphorylation (Figure 7A, D) as was observed in yeast cells (Figure 1D, E) suggesting that priming requirements for S303 phosphorylation may change in different expression systems.

## Discussion

Mammalian HSF1 activity is regulated via complex regulatory mechanisms that include post-translation modifications as well as inter- and intra-molecular protein-protein interactions [2]. While our understanding of these regulatory mechanisms remains incomplete, earlier work has suggested that many of these mechanisms may be conserved in yeast [25], [26], [27], [28]. This is evident, in part, by repression of the human HSF1 protein when it is expressed in *S. cerevisiae* via coiled-coil domain and HSF1 loop interactions. In this report we show that evaluation of the mechanisms that regulate HSF1 activity in yeast via post-translational modifications can lead to important insights into the mechanisms that regulate HSF1 in mammalian cells.

Previous experiments using HSF1 fusions with the constitutively bound Gal4 or lexA DNA-binding domains demonstrated that phosphorylation of S303 contributed to the repression of HSF1 transactivation [17], [18]. In this report we show that alanine substitution of S303, in the context of full length HSF1, also results in increased levels of trimerized HSF1 both in un-stressed yeast and in mammalian cells. This suggests that aside from repressing transactivation, S303 phosphorylation can also repress earlier points in the HSF1 activation pathway. Interestingly, we also show that repression of HSF1 activity through S303 phosphorylation may occur independent of K298 sumoylation in yeast, as arginine substitution of K298 does not promote HSF1 activation in yeast. It should be noted that not all of the mechanisms that regulate human HSF1 in mammalian cells are conserved in yeast. While human HSF1 is repressed in both yeast and mammalian cells through an amino-terminal coiled-coil as well as a carboxyl-terminal linker domain, the ability of wild type human HSF1 to respond to proteotoxic compounds or thermal stress, for example, appears to be strikingly absent in yeast [25], [27], [28]. Nevertheless, the ability of S303 phosphorylation to promote repression of human HSF1 in yeast independent of K298 sumoylation suggests that our understanding of the mechanisms by which S303 phosphorylation represses HSF1 activity remains incomplete. S303 and S307 are located in the regulatory domain of HSF1, a proposed binding site for the protein chaperone Hsp90 [41]. As such, it is tempting to speculate that phosphorylation of these residues might affect binding to Hsp90.

An understanding of how phosphorylation regulates HSF1 activity and what protein kinases phosphorylate HSF1 remains largely incomplete [20], [22]. Early reports showed that *in vitro*, HSF1 S307 phosphorylation acted as an essential priming event for S303 phosphorylation [22]. However, a subsequent report showed this priming event was not required *in vivo* and that HSF1 S303 phosphorylation occurred independent of S307 phosphorylation in K562 cells [16]. The work presented here using the yeast model system furthers our understanding of these regulatory mechanisms and may begin to clarify the conflicting mechanisms underlying S303 phosphorylation. Specifically, our data suggest that while phosphorylation of S303 can occur independently of S307 phosphorylation in both yeast and mammalian cells, S303 phosphorylation may be enhanced by S307 phosphorylation in the non-native yeast system. While a mechanistic basis for this difference in the requirements for S303 phosphorylation remains unknown when HSF1 is expressed in yeast, structural differences could change the priming requirements for S303 phosphorylation. Such changes in HSF1 might occur due to different protein interactions and as such it is not surprising that in *in vitro* experiments, using only recombinant HSF1 protein, phosphorylation of S303 is fully dependent on S307 phosphorylation. However, further studies will be required to fully test these hypotheses.

Here, we demonstrate that in both yeast and mammalian cells phosphorylation of HSF1 S303 appears to occur independently of GSK3, previously thought to be the primary kinase responsible for S303 phosphorylation [20], [22]. Rather, as suggested by loss of function analysis, we propose that the MAPK Slt2 is one candidate that phosphorylates HSF1 at S303 in yeast though residual phosphorylation of HSF1 at S303 in an *slt2*Δ strain suggests that other MAPKs may also contribute to S303 phosphorylation. Differences in HSF1 structure between the *in vivo* and *in vitro* systems may also explain why different kinases can target S303 for phosphorylation under different conditions. We speculate that under some cellular conditions, for example physiological stress or different cell types, HSF1 structure may be altered, thereby shifting the S303-kinase specificity from a MAPK to GSK3. This might, in part, contribute to the complexity in identifying all of the mammalian kinases that phosphorylate S303. While GSK3 does not appear to phosphorylate HSF1 at S303 *in vivo*, data presented here nevertheless support a role for GSK3 as a repressor of HSF1 activity. It should be noted that several other serine residues in the HSF1 coding sequence, including S307, are located within putative GSK3 consensus sites [39].

The importance in understanding HSF1 regulation is underscored by recent findings showing that pharmacological activation of HSF1 can increase protein chaperone expression and ameliorate cytotoxicity in models of protein folding disease [28], [42], [43], [44], [45]. As such, it is important to further our understanding of the mechanisms that repress HSF1 activity as potential points of therapeutic intervention in disease. For example, our data has shown that the loss of S303-dependent HSF1 repression can lead to the accumulation of protein chaperones and as such could be efficacious in the treatment of protein folding diseases. In support of this possibility Rimoldi *et al* showed that over-expression of the HSF1 S303G mutant in HeLa cells reduced aggregation and inclusion formation of an aggregation prone Ataxin1-31Q mutant protein [46] In addition, Fujimoto *et al* showed that overexpression of a constitutively active HSF1 mutant lacking the regulatory domain, which includes S303 and S307, suppressed the aggregation and cytotoxicity of a mutant Huntingtin protein in both cell culture and mice [47]. Furthermore, Carmichael *et al* suggested that GSK3-inhibitors might prove useful in the treatment of polyQ-expansion diseases [48].

## Materials and Methods

### Yeast Strains, Plasmids

*S. cerevisiae* strains used in this study are listed in Table 1. Yeast expression plasmids pRS424-GPD-HSF1 and pRS424-GPD-HSF1LZ4m were described previously [25]. Point mutations were introduced into the HSF1 coding sequence using the QuickChange Site-directed mutagenesis kit (Stratagene) and confirmed by DNA sequencing. YEp351-*Slt2-FLAG* was kindly provided by Dr. David E. Levin [49]. Mammalian expression plasmids were generated by subcloning the HSF1 open reading frame from yeast vectors into the mammalian vector pcDNA3.1.

**Table 1:** Yeast strains used in this study.

Strain	Genotype
PS145	*MAT*a *ade2-1 trp1-1 can1-100 leu2-3*, *112 his3-11,15 ura3-1 hsf1*Δ::*LEU2* Ycp50gal-*yHSF*
YPH499	*MAT*a *ura3-52 lys2-801 ade2-101 trp1-*Δ*63 his3-*Δ*200 leu2-*Δ*1*
BY4741	*MAT*a *his3*Δ*1 leu2Δ0 met15Δ0 ura3Δ0*
LNY1	*MAT*a *ade2-1 trp1-1 can1-100 leu2-3*, *112 his3-11,15 ura3-1 hsf1*Δ::*LEU2* Ycp50gal-*yHSF rim11Δ::HIS3*
LNY2	*MAT*a *ade2-1 trp1-1 can1-100 leu2-3*, *112 his3-11,15 ura3-1 hsf1*Δ::*LEU2* Ycp50gal-*yHSF slt2Δ::HIS3*
LNY3	*MAT*a *ura3-52 lys2-801 ade2-101 trp1-*Δ*63 his3-*Δ*200 leu2-*Δ*1 rim11Δ::TRP1 mck1Δ::HIS3 mrk1Δ::URA3 ygk3Δ::kanMX*

### Cell culture maintenance, transfection and siRNA

Mammalian cell lines used in the study were hsf1^−/−^ MEF cells [30] and HeLa cells (ATCC, CCL-2). The MEF cells were maintained in DMEM supplemented with 10% fetal bovine serum (FBS), 0.1 mM nonessential amino acids, 100 U/ml penicillin/streptomycin and 55 µM 2-mercaptoethanol. HeLa cells were maintained in DMEM supplemented with 10% FBS and 100 U/ml penicillin/streptomycin. MEF cells were transfected with HSF1 expressing plasmids using a Nucleofector (Lonza) and Nucleofector solution MEF2. siRNA was purchased from Dharmacon and 2 nmoles of each siRNA were transfected into HeLa cells using Dharmafect 1. Knock-down of proteins was assayed 72 h after siRNA transfection by immunoblot analysis.

### Complementation assays

Growth curve experiments were carried out in 96-well plates as described previously [28]. For spot assays yeast cells were grown overnight in galactose-containing medium to allow for expression of GAL1-yHSF and reseeded the following day at O.D._600_ = 0.2 and spotted on either galactose or dextrose supplemented growth media.

### Immunoblot and Crosslinking Analysis

Protein extracts were generated from yeast cultures using glass bead lysis in cell lysis buffer (25 mM Tris, 150 mM NaCl, 1% Triton X-100, 0.1% SDS, 1 mM EDTA) supplemented with protease inhibitors (Roche) and Halt phosphate inhibitor cocktail (Thermo Scientific Pierce). Proteins extracts were generated from mammalian cell culture using cell lysis buffer supplemented with protease and phosphatase inhibitors. Protein concentrations were quantified using the BCA assay and 80–100 µg of total protein was resolved by SDS-PAGE and transferred to a nitrocellulose membrane. HSF1 oligomerization was assessed using the amine-specific cross-linker ethylene glycol bis-succinimidyl succinate (EGS) (Pierce). Crosslinking analysis were carried out as described previously [28]. Antibodies used in this study were anti-phospho-S303(pS303) (ab47369, Abcam), anti-HSF1 [28], anti-Pgk1, anti-FLAG (M2, Sigma), anti-Hsp70 (C92, Stressmarq), anti-β-catenin (6B3, Cell Signaling), anti-GAPDH (6C5, Ambion) and anti-GSK3α/β (D75D3, Cell Signaling). Quantification of immunoblot data was done using Photoshop.
